# A novel *SLC12A3* homozygous c2039delG mutation in Gitelman syndrome with hypocalcemia

**DOI:** 10.1186/s12882-018-1163-3

**Published:** 2018-12-17

**Authors:** Wenjun Yang, Shaoli Zhao, Yanhong Xie, Zhaohui Mo

**Affiliations:** The Endocrinology Department of the Third Xiangya Hospital, Central South University, Tongzipo Road, Changsha, 410013 China

**Keywords:** Gitelman syndrome, *SLC12A3* gene mutation, Hypocalcemia

## Abstract

**Background:**

Gitelman syndrome (GS) is a rare autosomal recessive renal tubular disease, caused by mutations in the *SLC12A3* gene, which encodes the renal thiazide-sensitive Na/Cl cotransporter (NCCT) in the distal renal tubule.

**Case presentation:**

A 23-year-old woman was admitted with limb numbness, recurrent tetany and palpitation. Laboratory tests showed hypokalemic alkalosis, hypomagnesemia, hypocalcemia and secondary hyperaldosteronism, as well as hypocalciuria and transient decreased PTH. Next-generation sequencing detected a novel homozygous mutations c.2039delG in the *SLC12A3* gene, and her father and children were all heterozygous carriers.

**Conclusion:**

We reported a case of GS with a novel homozygous frame-shift mutation of *SLC12A3*, and reviewed recent literatures about diagnosis, differential diagnosis and treatments. Hypocalcemia in Gitelman syndrome is rare, and may be related to inhibited PTH secretion induced by hypomagnesemia.

**Electronic supplementary material:**

The online version of this article (10.1186/s12882-018-1163-3) contains supplementary material, which is available to authorized users.

## Background

Gitelman syndrome (GS) is an autosomal recessive disease, characterized by hypokalemic alkalosis, hypomagnesaemia, hypocalciuria, low blood pressure, first described by Gitelman in 1966 [1]. It is caused by mutations in the *SLC12A3* gene, which encodes the renal thiazide-sensitive Na/Cl cotransporter (NCCT) in the distal renal tubule [2]. Here, we report a case of a new mutation of *SLC12A3* in an Asian pedigree.

## Case presentation

A 23-year old female was hospitalized due to limb numbness, muscle weakness for one month, recurrent tetany and palpitation for 10 days. She visited the local hospital and was found to have a decreased plasma potassium (2.24 mmol/l) and calcium (1.6 mmol/L), while plasma magnesium was unknown because of limited conditions at the hospital. Her condition seem to have improved after treatment with “potassium chloride” and “calcium gluconate”, however, symptoms recurred after discharge, so she visited our hospital. She denied having any other diseases or taking any medication prior to her visit to the hospital. Her two children and her father were healthy without any similar symptoms. Her mother and her only sister however, had passed away at the age of 30 years old and 1 year old, respectively, with unclear diagnosis. Her parents were from the same town but denied of consanguineous marriage.

Physical examination on admission showed normal vital signs, except for her relatively low blood pressure, which fluctuated between 88 and 108/56-78 mmHg. She had a height of 158 cm and body weight of 46 kg. The lower limb muscle strength was decreased, accompanied by diminished tendon reflexes. The Trousseau’s sign was positive. Other signs of physical examinations were normal.

Laboratory test results are shown in Table 1.

**Table 1:** Clinical tests of the patient

Examination items	Test value		Reference value
Serum biochemicals			
Potassium (mmol/L)	3		3.5–5.3
Sodium (mmol/L)	142		137.0–147.0
Chloride (mmol/L)	99		99.0–110
Calcium (mmol/L)	2.3		2.2–2.7
Magnesium (mmol/L)	0.32		0.75–1.02
Phosphate (mmol/L)	1.39		0.85–1.51
CO2CP (mmol/L)	33.5		23–29
creatine (umol/l)	43		41–73
BUN (mmol/l)	3.15		2.6–7.5
Analysis of arterial blood gas			
PH	7.506		7.35–7.45
PO2 (mmHg)	102		90–110
PCO2 (mmHg)	35.1		35–45
HCO3 (mmol/L)	28.9		22–27
Urine test			
Urine specific gravity	1.01		1.010–1.025
PH	7.5		4.60–8.00
24-h urine			
Potassium (mmol/24 h)	89		51–100
Calcium (mmol/24 h)	0.25		2.5–7.5
Magnesium (mmol/24 h)	2.92		1.0–10.5
Phosphate (mmol/24 h)	9.44		16–42
Creatinine (mmol/24 h)	7.45		7–18
Calcium/creatinine (mmol/mmol)	0.03		> 0.2
RAAS system	Before high sodium^a^	After high sodium	
Plasma renin activity (ng /ml.h)	12.95	1.93	1.15–2.33
Angiotensin I (ng /ml)	16.45	3.76	0.07–1.5
Angiotensin II (pg /ml)	97.68	65.62	25–60
Aldosterone (pg / ml)	225.1	152.9	30–160
ARR (ng/dl:ng/ml.h)	1.74	7.92	< 20

High sodium load test^a^: the patient has intravenous infusion of 0.9% saline, at a rate of 500 ml/h for 4 h between 8:00 and 12:00 am, and takes blood tests of aldosterone, renin activity, angiotensin before and after infusion. In patients with GS or normal people, plasma aldosterone and renin activity will be inhibited, while in patients with primary aldosteronism, plasma aldosterone levels are not inhibited

Her thyroid function tests and glucocorticoid level were within the normal range, and autoimmune antibodies were negative, while the 25(OH)VD3 was 17.08 ng/mL (reference value: > 20 ng/ml). The PTH was 11.2 pg/ml (reference value: 15-65 pg/ml) at the local hospital, and returned to the normal range 2 days later. At our hospital the value was 32.66 pg/ml. Her chest X-ray and abdominal ultrasound were normal and the dual-energy x-rays showed osteopenia (T value was − 1.8). Laboratory results suggested hypokalemia, hypomagnesemia, hypocalciuria, metabolic alkalosis, and secondary hyperaldosteronism. These characteristics indicated GS. We also tested the serum electrolytes of the patient’s father and children, which showed normal results.

After obtaining informed consent from the patient and family members, genomic DNA was extracted from peripheral blood for *SLC12A3* and other related genes of Bartter syndrome whole genome sequencing in the Changsha Kingmed Center for Clinical Laboratory. Genomic DNA was extracted via centrifugal column method, with Qiagen kit. Gene sequencing was performed as referred [3].

**Fig. 1:**
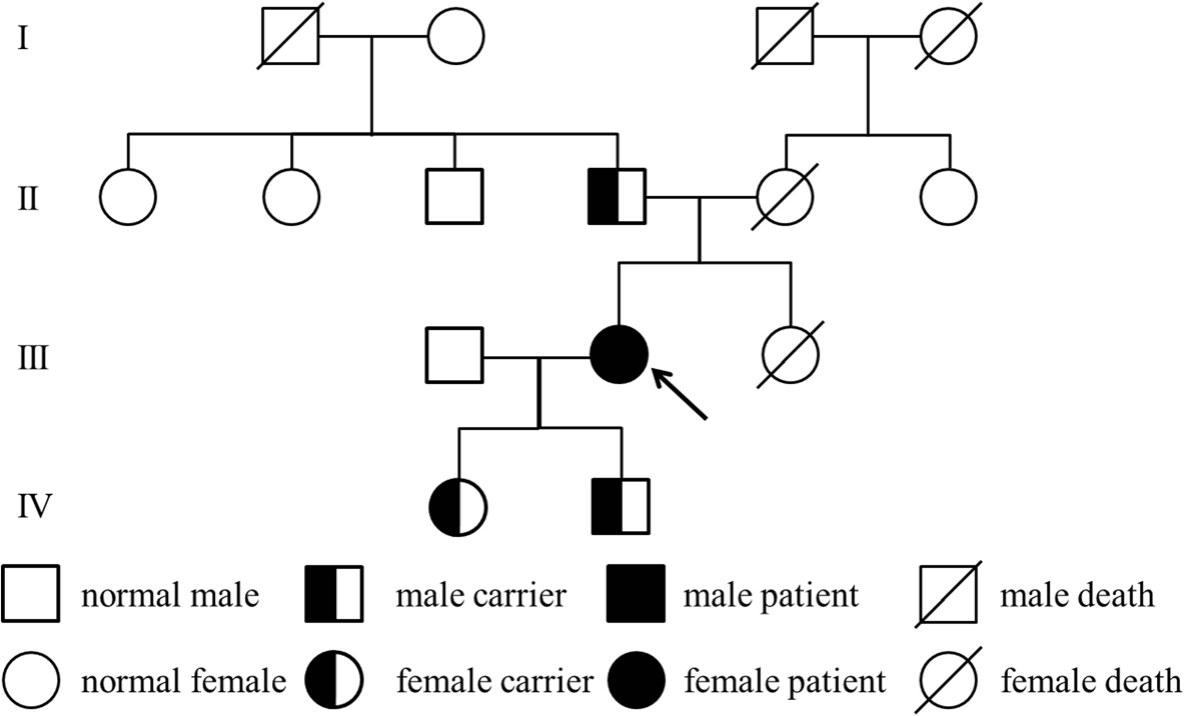
Pedigree analysis

**Fig. 2:**
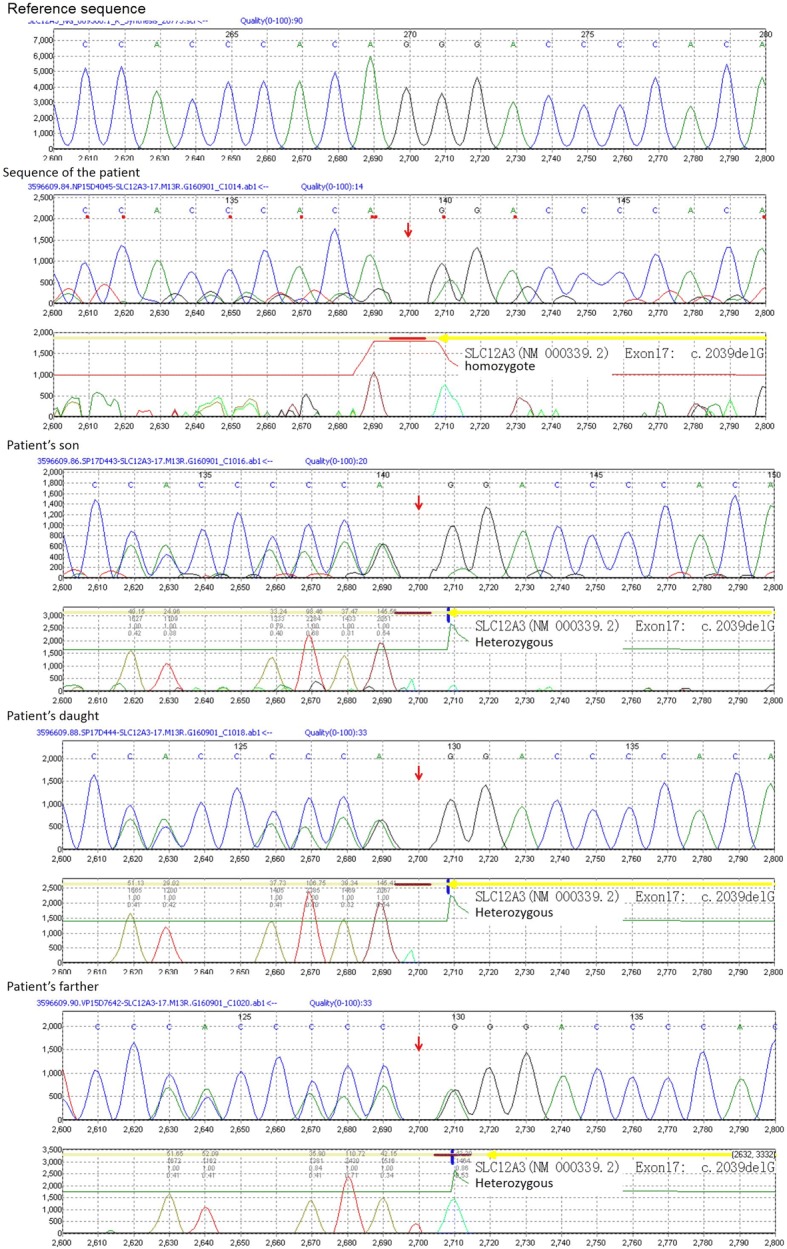
Sequences of the patient, her children and her father. The above sequence column is the reference sequence, next are the sequence of the patient and her son, daughter, father, respectively. The columns below tested sequences are subtracted results of tested sequence and reference sequence, which show homozygous c.2039delG of the patient and heterozygous mutation of her children and father)

We found that the patient was positive for *SLC12A3* gene homozygous mutation (exon 17: nucleic acid mutation: c.2039delG, amino acid mutation: p.Gly680AspfsTer20). It was a frameshift mutation in exon 17, which began from the glycine in the No.680, mutated into aspartate, and leading to premature termination of NCCT protein in the No.700 amino acid (terminator codon TGA). To exclude the possibility that the c2039delG is heterozygous mutation with deletion of the other allele at this locus, we performed copy number variation (CNV) analysis using next-generation sequencing data, the result is shown in Additional file 1: Figure S1, confirming the c.2039delG is bi-allelic homozygous mutation. A heterozygous mutation in the same site was observed in the sequences of the patient’s father and her two children. C.2039delG was a novel mutation and has not been reported in SNP databases such as Exome Aggregation Consortium and 1000Genome. We also tested *SLC12A1, KCNJ1, CLCNKB, BSND, CLCNKA/CLCNKB* related to Bartter syndrome, showing negative results. Pedigree analysis was shown in Fig. 1, and the sequence map is shown in Fig. 2.

## Discussion and conclusions

GS is an inherited renal tubulopathy with a prevalence estimated at one to ten per 40,000 people, and more prevalent in Asia [4]. Diagnosis of GS is based on the clinical symptoms, biochemical abnormalities and genetic test. Common symptoms include salt craving, cramps, muscle weakness, fatigue, etc. Fainting, polyuria, arthralgia, chondrocalcinosis, growth retardation, vertigo, ataxia, tetany, seizure, etc. can also occur [5]. Our patient had numbness, tetany, muscle weakness and palpitations, and low blood pressure. Her symptoms and typical laboratory examinations together corresponded to GS. The criteria for diagnosis of GS is identification of biallelic inactivating mutations in *SLC12A3* [5]. To date, more than 400 mutations of *SLC12A3* have been identified, including missense/ nonsense mutations, splicing, deletions, insertions, reading frame shifting mutation, etc. Missense mutations are the most frequent. According to a gene analysis of 54 Chinese GS patients by Ruijin Hospital and 42 patients by Peking Union Medical College Hospital, T60 M and D486N are the most common amino acid mutations in Chinese patients [6, 7], while IVS9 + 1G > T was reported as the most common mutation in a European population [8]. Frame shift mutations were much fewer. In this study, we describe this c.2039delG frame shifting mutation for the first time. The proband has homozygous mutations, and her father and two children are heterogeneous carriers. According to the autosomal recessive inheritance rules, the proband’s mother at least carried a heterogeneous mutation.

The differential diagnosis of GS includes diuretic, specifically thiazide abuse and chronic vomiting. Additionally some disorders affecting the kidneys or the gastrointestinal tract can also result in hypokalemic metabolic alkalosis with hyperreninemic secondary aldosteronism. It has been reported that GS-like manifestations could be a rare complication of cisplatin [9]. Autoimmune diseases, Sjögren syndrome, for example, was reported to cause renal tubular disorders, and cause typical features of GS [10–12]. Among various differential diagnosis, classical Bartter syndrome (type III) is the most indistinguishable, though it usually has early onset, and normal plasma magnesium. However, Mutations in the HNF1β gene can mimic the electrolyte abnormalities of GS, approximately 50% of patients develop hypomagnesemia because of renal magnesium wasting, often accompanied by hypocalciuria [13]. Therefore genetic test is crucial. According to the consensus and guidance from KDIGO, the use of a next generation sequencing–based gene panel to parallel sequence all genes that are relevant in the differential diagnosis of GS is recommended [5]. Besides, GS can be accompanied with other diseases, making differential diagnosis difficult. In some GS cases, thyrotoxicosis coexsits [14]. Although Sjögren syndrome can cause GS-like symptoms, sometimes they may be comorbidities [15], again making gene sequencing much more important.

Hypocalcemia was rarely reported in GS cases, because calcium excretion through urine is very little. Some explanation to hypocalcemia is that hypomagnesaemia causes defective cyclic AMP generation in the parathyroid glands and in the PTH target organs, leading to impaired secretion of PTH as well as end-organ resistance to PTH [16]. Furthermore PTH could increase serum magnesium by increasing its gastrointestinal absorption and bone resorption [17]. This may explain why our patient had hypocalcemia and transiently decreased PTH. The patient regained normal calcium and PTH status soon. It also reminded us that her tetany was mainly due to hypomagnesemia because it recurred at a normal plasma calcium level.

Treatment of GS is usually managed by a liberal salt (NaCl) intake, together with oral magnesium and potassium supplements. The recommended target for potassium is 3.0 mmol/l and magnesium 0.6 mmol/l. We treated this patient with chloride (KCl), potassium magnesium aspartate. Considering her refractory hypokalemia and recurrent tetany, we added spironolactone and indomethacin, a nonselective inhibitor of cyclooxygenase (COX). Indomethacin is rarely used in GS, because plasma prostaglandin E2 (PGE2) levels in GS are usually normal. But studies found that significantly higher PGE2 and PGE2 metabolites (PGEM) levels in GS patients’ urine and PGEM in their plasma. Also, higher urinary PGEM levels indicated more severe clinical manifestations and NCC dysfunction [18]. There was study compared the efficacy and safety of 75 mg indomethacin, 150 mg eplerenone, or 20 mg amiloride added to constant potassium and magnesium supplementation in GS patients, and the result showed each drug increased plasma potassium level by approximately 0.3 mmol/l [19]. But for long-term treatment, the gastrointestinal, cardiovascular side effects and nephrotoxicity should be considered. After two years follow-up, the patient had a reasonable potassium level of 3.1–3.5 mmol/l, and her symptoms were alleviated, while the plasma magnesium level was still unstable, between 0.4 and 0.8 mmol/l. It is more difficult to supplement magnesium as magnesium salts frequently cause diarrhoea. Among organic magnesium salts, MgCl_2_ is preferred, which also compensates the urinary loss of chloride. However, it is unavailable in many places therefore a more readily available therapy should be researched.

In summary, we reported a GS case with hypocalcemia with a novel c.2039delG mutation of *SLC12A3* gene with typical clinical symptoms and laboratory findings. Genetic testing makes the diagnosis easier but the cost remains expensive. A better treatment regimen is still worthy to work on.

## Additional file

Additional file 1: **Figure S1.** The second generation sequencing of the patient, showing c.2039delG homozygous mutation of the patient. (TIF 607 kb)

## Abbreviations

COX: Cyclooxygenase
GS: Gitelman syndrome
NCCT: Na/Cl cotransporter
PGE2: Prostaglandin E2
PGEM: Prostaglandin E metabolites
PTH: Parathyroid hormone
