# Supplementary figures and images for: A novel SLC12A3 homozygous c2039delG mutation in Gitelman syndrome with hypocalcemia
Source: BMC Nephrol. 2018 Dec 17;19:362. doi: 10.1186/s12882-018-1163-3 (PMC6296056; doi:10.1186/s12882-018-1163-3)

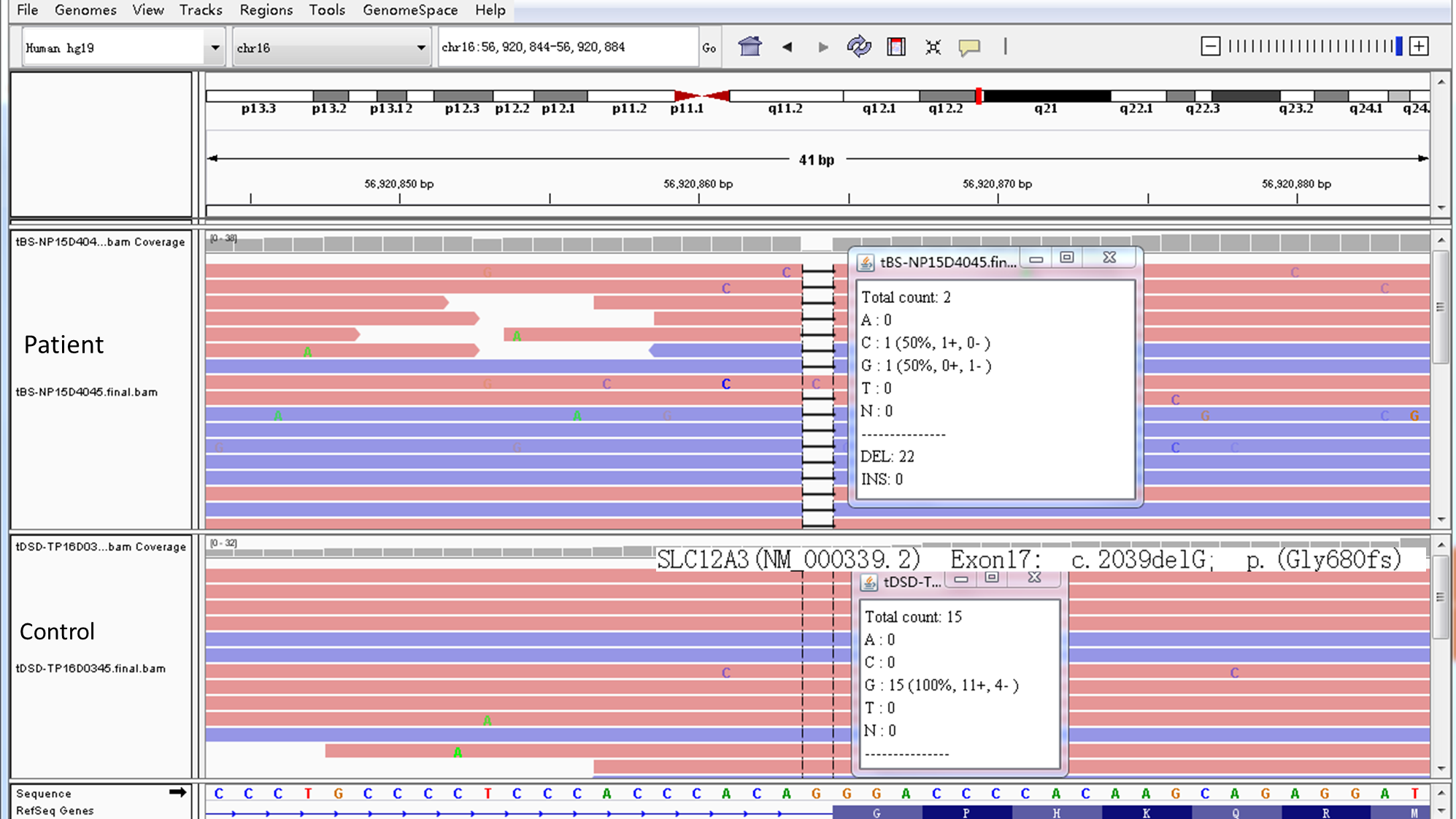

Supplement: Supplementary file 1 — Figure S1. The second generation sequencing of the patient, showing c.2039delG homozygous mutation of the patient. (TIF 607 kb) [file 12882_2018_1163_MOESM1_ESM.tif]
